# Emergency Department Patient Satisfaction with Treatment of Low-risk Pulmonary Embolism

**DOI:** 10.5811/westjem.2018.9.38865

**Published:** 2018-10-18

**Authors:** Laura E. Simon, Hilary R. Iskin, Ridhima Vemula, Jie Huang, Adina S. Rauchwerger, Mary E. Reed, Dustin W. Ballard, David R. Vinson

**Affiliations:** * Kaiser Permanente, Division of Research, Oakland, California; † University of Michigan Medical School, Ann Arbor, Michigan; ‡ University of Cincinnati College of Medicine, Cincinnati, Ohio; § Kaiser Permanente San Rafael Medical Center, Department of Emergency Medicine, San Rafael, California; ¶ Kaiser Permanente Sacramento Medical Center, Department of Emergency Medicine, Sacramento, California

## Abstract

**Introduction:**

Many emergency department (ED) patients with acute pulmonary embolism (PE) who meet low-risk criteria may be eligible for a short length of stay (LOS) (<24 hours), with expedited discharge home either directly from the ED or after a brief observation or hospitalization. We describe the association between expedited discharge and site of discharge on care satisfaction and quality of life (QOL) among patients with low-risk PE (PE Severity Index [PESI] Classes I–III).

**Methods:**

This phone survey was conducted from September 2014 through April 2015 as part of a retrospective cohort study across 21 community EDs in Northern California. We surveyed low-risk patients with acute PE, treated predominantly with enoxaparin bridging and warfarin. All eligible patients were called 2–8 weeks after their index ED visit. PE-specific, patient-satisfaction questions addressed overall care, discharge instruction clarity, and LOS. We scored physical and mental QOL using a modified version of the validated Short Form Health Survey. Satisfaction and QOL were compared by LOS. For those with expedited discharge, we compared responses by site of discharge: ED vs. hospital, which included ED-based observation units. We used chi-square and Wilcoxon rank-sum tests as indicated.

**Results:**

Survey response rate was 82.3% (424 of 515 eligible patients). Median age of respondents was 64 years; 47.4% were male. Of the 145 patients (34.2%) with a LOS<24 hours, 65 (44.8%) were discharged home from the ED. Of all patients, 89.6% were satisfied with their overall care and 94.1% found instructions clear. Sixty-six percent were satisfied with their LOS, whereas 17.5% would have preferred a shorter LOS and 16.5% a longer LOS. There were no significant differences in satisfaction between patients with LOS<24 hours vs. ≥24 hours (p>0.13 for all). Physical QOL scores were significantly higher for expedited-discharge patients (p=0.01). Patients with expedited discharge home from the ED vs. the hospital had no significant difference in satisfaction (p>0.20 for all) or QOL (p>0.19 for all).

**Conclusion:**

ED patients with low-risk PE reported high satisfaction with their care in follow-up surveys. Expedited discharge (<24 hours) and site of discharge were not associated with differences in patient satisfaction.

## INTRODUCTION

There is increasing evidence that it is safe and effective to discharge home emergency department (ED) patients with acute pulmonary embolism (PE) at low risk of short-term adverse events, determined using a validated risk score or outpatient exclusion criteria.^1^–^4^ The Pulmonary Embolism Severity Index (PESI) is a validated prognostic tool that can be used to stratify PE patients by risk of 30-day, all-cause mortality^1^,^5^,^6^ and help identify eligible candidates for outpatient management. The PESI categorizes patients into five ascending risk classes, with many patients in Classes I–III eligible for outpatient management.^7^,^8^

While home treatment of PE has been shown to be safe and effective, rates of outpatient management vary widely,^9^–^12^ and little is known about patient satisfaction with care and health-related quality of life (QOL) when managed at home. Health-related QOL refers to an individual’s perception of their health and the effect it has on his or her daily life.^13^ Recent research has found that patients treated for isolated deep vein thrombosis (DVT) at home with low-molecular-weight heparin report QOL scores similar to those treated as inpatients, but with better social functioning.^14^,^15^ Limited research has been conducted focusing exclusively on patients with PE, although existing research indicates that outpatient management of PE has been satisfactory.^1^,^16^,^17^ Patients with other conditions, such as community-acquired pneumonia and stroke, have also reported comparable or improved satisfaction and QOL scores following outpatient management, compared to inpatient treatment.^18^–^20^ However, to our knowledge, little has been done to examine the effects of length of stay (LOS) within a population of low-risk PE patients. 1

This telephone survey study of patients with objectively-confirmed PE within 21 community EDs examined patient satisfaction with care and QOL following their index ED visit. To understand the impact of different treatment pathways on patients with low-risk PE, we compared care satisfaction and QOL scores between patients with expedited home discharge (LOS<24 hours) and those without (LOS≥24 hours). Furthermore, we sought to determine any differences in satisfaction due to site of discharge, either from the ED or from the hospital, for those with a short LOS. We hypothesized that patients discharged within 24 hours would report similar, if not improved, satisfaction with care and QOL following their ED visit and that satisfaction ratings would not be greatly affected by discharge location.

## METHODS

### Study Design, Setting, and Population

This telephone-based survey of patients two weeks after an ED diagnosis of acute PE was undertaken in Kaiser Permanente (KP) Northern California, a large, integrated healthcare delivery system that provides comprehensive medical care for more than four million members. KP members represent approximately 33% of the population in areas served and are highly representative of the surrounding population.^21^ KP Northern California is supported by a comprehensive integrated electronic health record (EHR) (Epic, Verona, Wisconsin) fully deployed in 2009. 8 The study was approved by the KP Northern California Institutional Review Board (IRB).

**Population Health Research Capsule**

What do we already know about this issue?

Home management of pulmonary embolism (PE) is safe and effective for select low-risk patients. Little is known about patient care satisfaction or quality of life.

What was the research question?

Did length of stay (LOS) or discharge disposition impact patients’ satisfaction with care or quality of life?

What was the major finding of the study?

Patient care satisfaction was high. Physical quality of life was higher for those with a length of stay <24 hours.

How does this improve population health?

Improved understanding of PE patients’ care satisfaction and quality of life can help physicians in the development of care strategies.

This patient survey was a component of a multicenter, retrospective cohort study of ED patients with acute, objectively-confirmed PE. The MAPLE study – Management of Acute Pulmonary Embolism – was undertaken at 21 non-rural community EDs from January 2013 through April 2015 and has been described elsewhere.^8^,^22^ Management of patients with acute PE during the study period commonly included warfarin with 5–7 days of bridging with enoxaparin. Direct oral anticoagulants were not commonly used at the time.

**Figure 1:**
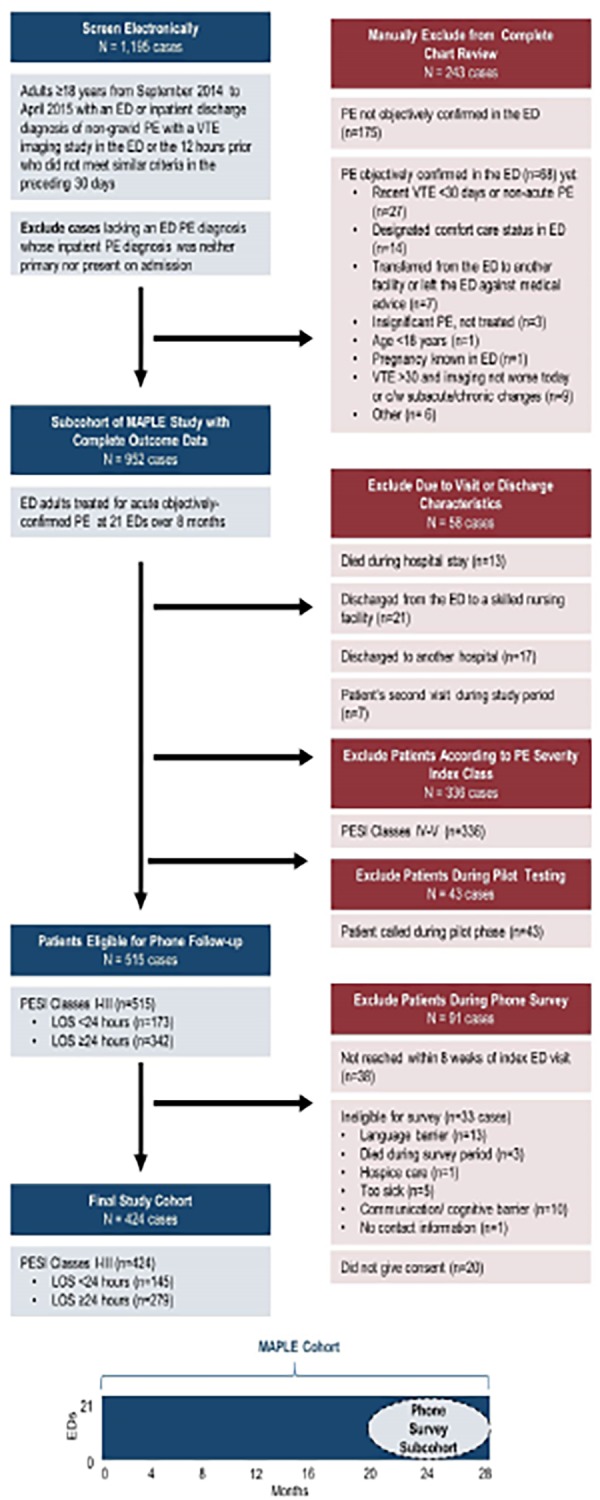
Cohort assembly of emergency department patients with acute pulmonary embolism for telephone follow-up survey. *ED*, emergency department; *PE*, pulmonary embolism; *VTE*, venous thromboembolism; *C/w*, consistent with; *MAPLE*, Management of Acute PuLmonary Embolism study; *PESI*, Pulmonary Embolism Severity Index; *LOS*, length of stay.

We depict the cohort assembly for the MAPLE study in Figure 1. We undertook the patient survey during the final eight months of the MAPLE study to coincide with the intervention arm of a controlled, pragmatic study to evaluate the impact of electronic clinical decision support on site-of-care decision-making for ED patients with acute, objectively-confirmed PE (the eSPEED study – electronic Support for Pulmonary Embolism Emergency Disposition). 9 Patients who met criteria for the MAPLE study from September 2014 through April 2015 were eligible for the telephone-based survey if they were classified as PESI Classes I–III. For site-of-care analysis, we defined hospitalization to include admission to inpatient services as well as admission to ED-based, short-term (<24 hours) outpatient observation units.

We identified patients for the telephone survey in the following manner: Each week, the study programmer analyst obtained data for patients with a recent ED visit who appeared to be eligible for the survey based on ED/inpatient discharge diagnoses and evidence of radiological imaging for DVT or PE. A study investigator then reviewed these patients’ charts to determine if the ED visit was eligible for the study and to assess for exclusion criteria as described previously.^8^,^22^ A research assistant (RA) then reviewed the charts to evaluate for secondary exclusion criteria. Patients were excluded at this point if they were discharged from the ED to a skilled nursing facility, died in the ED, or were PESI Classes IV–V.

We chose to stratify by patient LOS, rather than site of treatment, because there is limited research about LOS effects on patient satisfaction. A 24-hour end-point was used for our definition of an expedited discharge as this would include patients discharged directly home from one of our EDs (median LOS approximately 5.4 hours) 8 as well as a majority of those discharged home from a short-term observation unit. Such a time frame is similar to that used in other prospective studies of outpatient PE management.^1^,^2^,^17^,^23^ Two RAs contacted eligible patients for telephone interviews. To communicate directly with patients who were hard of hearing, the California Relay Service line was used. We excluded patients who could not complete the survey due to English proficiency level, cognitive impairment, or debility. Patients in PESI Classes I–III who consented to the survey within eight weeks of their index ED visit constituted our final cohort (Figure 1).

### Phone Survey Development and Script

The follow-up phone survey was intended to evaluate patient site-of-care preferences, satisfaction with treatment, and QOL following discharge. The PE-specific, patient-satisfaction questions were adapted from Aujesky et al. and modified for relevance and clarity. 1 Questions asked about satisfaction with overall care, discharge instruction clarity, and LOS. To assess patient QOL, we adapted questions and protocol from the eight-item Short Form Health Survey to meet the needs of a phone-based, interviewer-assisted QOL survey. Our survey assessed eight aspects of health-related QOL, summarized as physical and mental scores.^13^

The phone survey instrument was pre-tested to assess length and clarity of wording and piloted with eligible participants prior to the study start date. Pilot testing identified minor wording changes that were needed for clarity and decreased the number of questions for redundant concepts, resulting in 11 multiple-choice questions. The final text of the survey was approved by the study team and was used for the duration of the study (Appendix). The survey script included IRB-approved language requesting informed consent to participate in the phone survey.

### Phone Survey Administration

Two RAs were trained and overseen by the study investigators and the study project manager; weekly meetings were held to address survey administration difficulties and to ensure compliance with survey protocol. The RAs conducted phone surveys with eligible patients starting 12–14 days after the index ED visit; potential participants were not contacted until they had been discharged home from any inpatient stay. Attempts to contact potential participants occurred between 8 a.m. and 9 p.m. seven days a week, with a maximum of 15 outreach attempts. Outreach ceased if a participant was determined to be ineligible, refused to participate, or eight weeks had passed since their index ED visit, whichever occurred first. Survey responses were recorded using paper data sheets or a customized, online survey form. A trained study RA later entered data from paper data sheets into the online form.

### Statistical Analysis

Analysis included univariate and bivariate descriptive statistics, and examined differences between patients with expedited discharge and those admitted for ≥24 hours. Responses to the overall satisfaction and instruction clarity questions were condensed for statistical analysis. For overall care, we dichotomized responses into two categories: satisfactory/very satisfactory vs. neutral/unsatisfactory/very unsatisfactory. For instruction clarity we analyzed two categories: mostly clear/completely clear vs. mostly unclear/very unclear. LOS satisfaction was compared using three analyses: preferred shorter vs. satisfied/preferred longer, satisfied vs. preferred shorter/preferred longer, and preferred longer vs. satisfied/preferred shorter. We used chi-square test to examine the association between patient care satisfaction and LOS for all patients, and patient care satisfaction and site of discharge for those discharged within 24 hours. We also examined physical and mental QOL scores across patient stratifications using the Wilcoxon rank-sum test.

## RESULTS

Of all 1,195 low-risk PE patients electronically identified from the MAPLE study, 515 patients were eligible for the phone survey and called by interviewers (Figure 1); 424 completed the follow-up survey (response rate 82.3%). The median age of respondents was 64 years, 201 (47.4%) were male, and 145 (34.2%) had a LOS<24 hours. The median LOS for all patients was 36.1 hours, with a median of 14.3 hours in the expedited discharge cohort and 53.1 hours in the longer LOS cohort. The median time from ED arrival to survey completion was 16 days (interquartile range [IQR] [14–21] days). Additional patient characteristics are described in the Table.

**Figure 2:**
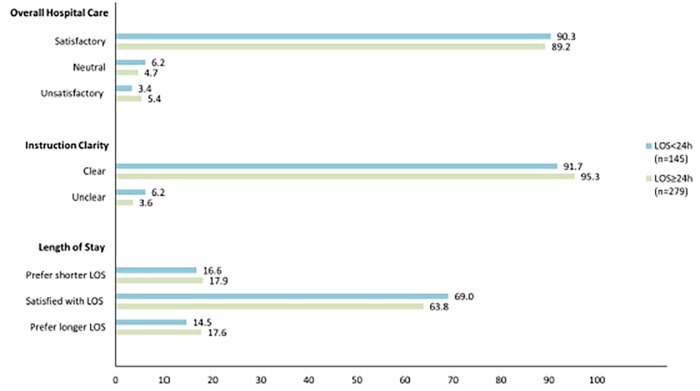
Responses to satisfaction questions by patients with low-risk pulmonary embolism, stratified by length of stay (LOS). Note: There were no significant differences in satisfaction rates between patients with a LOS<24 hours and a LOS≥24 hours (p>0.13 for all).

**Figure 3:**
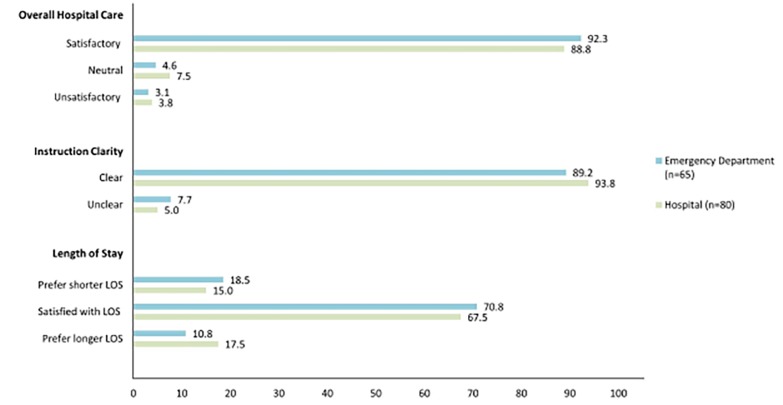
Responses to satisfaction questions by patients with low-risk pulmonary embolism, stratified by site of discharge. *LOS*, length of stay. Note: There were no significant differences in satisfaction rates between patients discharged from the emergency department and the hospital (p>0.20 for all).

We outline respondent answers to care satisfaction questions in Figure 2. There were no significant differences in scores between patients with a LOS<24 hours and ≥24 hours (p>0.13 for all). Collectively, 89.6% were satisfied with their overall care and 94.1% found instructions clear. The majority of patients were satisfied with their LOS (65.6%), although 17.5% would have preferred a shorter LOS and 16.5% a longer LOS. Of those discharged within 24 hours, 65 (44.8%) were discharged home from the ED and 80 (55.2%) from the hospital (response rates described in Figure 3). Patients discharged home directly from the ED vs. the hospital had no statistically significant differences in scores for overall care satisfaction (p=0.47), instruction clarity (p=0.33), or LOS satisfaction (p=0.67).

**Figure 4:**
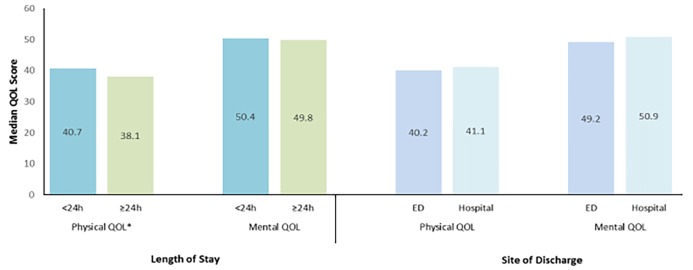
Physical and mental quality of life scores of patients with low-risk pulmonary embolism. *ED*, emergency department; *QOL*, quality of life. *No statistically significant differences found in patient QOL comparisons except for physical QOL when stratified by patient length of stay (p=0.01).

Physical and mental QOL stratified by LOS and site of discharge are represented in Figure 4. Patient physical QOL was significantly higher for patients discharged within 24 hours compared to those with a LOS≥24 hours (p=0.01). Mental QOL was not significantly different between LOS cohorts (p=0.69). When considering site of discharge for patients with a LOS<24 hours, QOL scores were not found to be significantly statistically different for physical (p=0.81) or mental (p=0.19) QOL.

## DISCUSSION

This telephone-based survey of low-risk PE patients discharged from 21 community medical centers describes patient satisfaction with care and QOL following their index ED visit. Patients reported high overall satisfaction (89.6%) and perception of instruction clarity (94.1%) for all treatment categories.

Patients of PESI Classes I–III were stratified by LOS and analyzed using a 24-hour cutoff. Satisfaction with overall care, clarity of instructions, and LOS did not significantly vary between patient groups. Aujesky et al. also described a similarity in the percentage of patients satisfied with their medical care between those with expedited discharge and a longer LOS, 1 reported in our study to be 90.3% and 89.2% (p=0.73), respectively. This analysis by Aujesky et al. was restricted to patients of PESI Classes I–II, whereas we expanded the eligible population to include patients in Class III. This decision was based on recent PE studies that found many Class III patients are eligible for outpatient care.^7^,^8^

Furthermore, our high level of patient satisfaction with overall care in the expedited discharge cohort is comparable to satisfaction ratings of outpatient management found in other studies on PE and venous thromboembolism, reported to be 91–92%.^1^,^24^ The satisfaction ratings reported in our study may also be improved by the use of exclusive oral anticoagulant treatments instead of the bridging subcutaneous enoxaparin injections required at the time of the survey.^25^ Ratings of instruction clarity were high in both the expedited discharge and longer LOS cohorts, 91.7% and 95.3%, respectively, and align with previously reported values.^24^ Of note, our physicians used templated discharge instructions that typically included the following: general patient education on PE, anticoagulation medication information, follow-up arrangements with their primary care provider and with Anticoagulant Services, and indications to seek medical care. The satisfaction ratings reported by those with a LOS<24 hours, and their similarity to those with a longer LOS, demonstrate that expedited discharge may not negatively impact the patient experience.

Among patients in PESI Classes I–III with LOS<24 hours, we did not detect any variation between those discharged from the ED and those admitted for a short hospital stay in any of the primary outcomes: overall care satisfaction, instruction clarity, or LOS satisfaction. The similarity between cohorts indicates that admission to the hospital or an observation unit is not required for patients to be highly satisfied with their care. Discharge directly from the ED was not shown to adversely affect a patient’s care experience.

Patient physical QOL 2–8 weeks after ED or hospital discharge was significantly higher in those with a LOS<24 hours compared to those with a LOS≥24 hours. There was no statistical difference in mental QOL between LOS cohorts. These findings are supported by other studies of outpatient management of DVT that have examined QOL in more detail. These studies have found no significant differences between treatment groups, except that patients treated at home score higher on physical and social functioning scales.^14^,^15^ Additionally, expedited discharge has been shown to not compromise QOL for eligible patients with conditions such as pneumonia, respiratory infection, and stroke.^18^–^20^ Furthermore, no statistically significant difference in QOL was found between patients discharged directly from the ED vs. those discharged from an observation unit or the hospital with a LOS<24 hours.

Our assessment of patient satisfaction with LOS demonstrates a potential area for improvement of patient care. Although the majority of patients were satisfied with their LOS, 17.5% would have preferred a shorter LOS (16.6% with LOS<24 hours and 17.9% with LOS≥24 hours, p=0.73) and 16.5% would have preferred a longer LOS (14.5% with LOS<24 hours and 17.6% with LOS≥24 hours, p=0.42). Other studies have also reported a similarly low incidence of patient dissatisfaction with LOS for PE treatments; Aujesky et al. found 14% of outpatients would have preferred a longer LOS and 29% of inpatients would have preferred to be treated at home.^1^ In our study, the mean LOS for patients discharged within 24 hours was 13.1 hours and for patients discharged after 24 hours was 72.1 hours, compared to the mean LOS in the study by Aujesky et al.: 25.9 hours for outpatient management and 106.9 hours for inpatient management.

These reported differences in mean LOS may explain the discontinuities in the proportion of patients that would have preferred earlier discharge between the two studies. Because we did not ask patients to explain their rationale for their LOS preferences, we can only conjecture about their reasoning. Possible explanations for preferring a shorter LOS may include improvement of symptoms early in the ED or hospital stay or the presence of obligations the patient did not want to miss due to being in the ED or hospital. Possible explanations for preferring a longer LOS include persistent symptoms after discharge, anxiety about early discharge, or prior expectations of the need for a longer stay.

Provider communication to set care expectations could help to improve the satisfaction we observed with LOS. Effective provider communication has been shown to increase patient satisfaction with care and their treatment compliance.^26^–^28^ Increased communication regarding the patient’s treatment needs, including the most appropriate site of care, the probable LOS, and the treatment end-points, could help in setting more realistic patient expectations and may increase patient satisfaction with LOS.

Although patient satisfaction and QOL were high in our community setting, there are limitations to the feasibility of expedited discharge and home management of patients with PE. Specific system requirements are necessary for safe and satisfactory discharge of PE patients, such as the ability to adequately select patients for home discharge, patient access to a follow-up care team, and lack of other indications for hospitalization.^8^,^9^ Additionally, although the overall treatment cost is lower for outpatient management,^29^,^30^ the cost burden of outpatient medication on the patient and their access to pharmacotherapy must be considered. 25 Assessment of these care aspects and patient preference should be incorporated into the site-of-care calculus.

## LIMITATIONS

This study has several limitations. First, this satisfaction and QOL survey was conducted over the telephone. Potential selection bias is introduced in that not all eligible participants could be reached within the first eight weeks following their index ED visit and some refused to respond to the survey. However, it would be expected that this selection bias would affect all patient groups equally, thus minimizing the effect on the overall comparison. There is also potential variation in patient responses due to the length of time between their index ED visit and their telephone survey. However, 75% of surveys were completed within the first three weeks of the index ED visit. Notably, a common hospital inpatient satisfaction survey, the Hospital Consumer Assessment of Healthcare Providers and Systems (HCAHPS), is administered between 48 hours and six weeks following discharge. 31 Some studies of expedited discharge of ED PE patients have conducted their patient-satisfaction surveys at 90 days.^17^ There are also potential generalizability limitations due to the exclusion of non-English speakers. Additionally, the effects of differences in select patient characteristics between the cohorts could be further analyzed.

During the time of this study, warfarin was the oral anticoagulant predominantly used for treatment of acute PE. However, there has since been a migration to the use of direct oral anticoagulants for patients with PE. While the effects of this change in pharmacotherapy on patient satisfaction are unknown, studies suggest that patients receiving these newer agents will have maintained, or even improved, levels of patient satisfaction.^25^

Because this study was conducted following an ED visit for objectively-confirmed PE, patient QOL was not assessed prior to PE diagnosis; thus, we could not adjust for QOL preceding the index ED visit. Also, although modified for our patient population, our QOL survey was not PE-specific nor as extensive as other health-related QOL surveys. While the limited number of questions affects our ability to comment on specific health-related domains, this survey was chosen because it was less time consuming for respondents and we sought to limit the burden on the patient and increase the response rate. Finally, overall health prior to ED arrival could not be accounted for and those with worse overall health may have been more likely to be hospitalized for over 24 hours. It is unknown how this may have affected patient satisfaction with care, but analysis of select comorbidities revealed similar rates between cohorts.

## CONCLUSION

In this telephone-based survey of ED patients with objectively-confirmed, low-risk acute PE, a high percentage reported satisfaction with their overall medical care and found discharge instructions clear. Additionally, the majority of patients were satisfied with their LOS. There were no statistically significant differences in patient-reported satisfaction between patients discharged within 24 hours vs. those with a LOS≥24 hours or between shorter LOS patients discharged directly from the ED vs. those admitted for a short hospital stay. The only significant difference in health-related QOL was a higher reported physical QOL for patients with a LOS<24 hours compared to patients with a LOS≥24 hours. These results may help inform future work to optimize site-of-care decision-making in patients with acute PE discharged from the ED or after a short observation or hospitalization.

## Supplementary Information



## Figures and Tables

**Table 1:** Clinical characteristics of emergency department patients with acute pulmonary embolism, stratified by patient length of stay (n = 424).

Patient characteristics	ED patient length of stay			

	LOS<24 hours N=145		LOS≥24 hours N=279	
	
	No.	%	No.	%
Age median (IQR), years	64 (50–76)		64 (52–76)	
LOS median (IQR), hours	14.3 (5.8–20.5)		53.1 (37.2–94.5)	
Sex, male	67	46.2	134	48.0
Comorbidities				
Cancer (history of or active)	34	23.4	76	27.2
Chronic lung disease (includes asthma)	44	30.3	86	30.8
Heart failure (diastolic or systolic)	17	11.7	30	10.8
Vital signs*				
Systolic blood pressure, mm Hg				
<100 and ≥90	21	14.5	30	10.8
<90	6	4.1	12	4.3
Pulse, beats/min				
≥100 and <110	30	20.7	43	15.4
≥110	48	33.1	84	30.1
Respiratory rate, breaths/min				
≥24 and <30	42	29.0	75	26.9
≥30	18	12.4	25	9.0
Oxygen saturation, %				
<94 and ≥90	40	27.6	53	19.0
<90	17	11.7	40	14.3
Temperature <36°C (96.8°F)	1	0.7	2	0.7
Altered mental statusŦ	1	0.7	2	0.7
Pulmonary Embolism Severity Index class				
I	53	36.6	77	27.6
II	56	38.6	110	39.4
III	36	24.8	92	33.0

*ED*, emergency department; *LOS*, length of stay; *IQR*, interquartile range.

* We report the most abnormal value in the direction in question. Vital signs include pre-arrival values from out-of-hospital and outpatient clinic settings if these were documented by the emergency physician. The numbers of missing vital signs were as follows: systolic blood pressure, n=2 (0.5%); pulse, n=2 (0.5%); respiratory rate, n=3 (0.7%); oxygen saturation, n=2 (0.5%); temperature, n=17 (4.0%).

Ŧ Altered mental status as defined by the Pulmonary Embolism Severity Index includes disorientation, lethargy, stupor, and coma.
