# Supplementary material for: Emergency Department Patient Satisfaction with Treatment of Low-risk Pulmonary Embolism
Source: West J Emerg Med. 2018 Oct 18;19(6):938–46. doi: 10.5811/westjem.2018.9.38865 (PMC6225929; doi:10.5811/westjem.2018.9.38865)
Supplement: Supplementary file 1 [file wjem-19-938-s001.pdf]

Appendix 1: Survey of Patients with Low-risk Pulmonary Embolism

1. Overall, how would you rate your health since your ER visit on [date]?
   ○ Excellent ○ Very good ○ Good ○ Fair ○ Poor ○ Very poor

2. Since your ER visit on [date], how much did physical health problems limit your usual physical activities (such as walking or climbing stairs)?
   ○ Not at all ○ Very little ○ Somewhat ○ Quite a lot ○ Could not do physical activities

3. Since your ER visit on [date], how much difficulty did you have doing your daily work, both at home and away from home, because of your physical health?
   ○ None at all ○ A little bit ○ Some ○ Quite a lot ○ Could not do daily work

4. How much bodily pain have you had during the time since your ER visit on [date]?
   ○ None ○ Very mild ○ Mild ○ Moderate ○ Severe ○ Very severe

5. Since your ER visit on [date], how much energy did you have?
   ○ Very much ○ Quite a lot ○ Some ○ A little ○ None

6. Since your ER visit on [date], how much did your physical health or emotional problems limit your usual social activities with family or friends?
   ○ Not at all ○ Very little ○ Somewhat ○ Quite a lot ○ Could not do social activities

7. Since your ER visit on [date], how much have you been bothered by emotional problems (such as feeling anxious, depressed or irritable)?
   ○ Not at all ○ Slightly ○ Moderately ○ Quite a lot ○ Extremely

8. Since your ER visit on [date], how much did personal or emotional problems keep you from doing your usual work, school or other daily activities?
   ○ Not at all ○ Very little ○ Somewhat ○ Quite a lot ○ Could not do daily activities

9. How would you rate your overall hospital care for this episode of blood clot in your lung?
   ○ Very satisfactory ○ Satisfactory ○ Neutral ○Unsatisfactory ○ Very unsatisfactory

10. When you went home from the hospital, you were given instructions for medical care and follow-up. Did you feel those instructions were…
    ○ Completely clear ○ Mostly clear ○ Mostly unclear ○Very unclear

11. How do you feel about the length of time that you stayed in the hospital?
    ○ Prefer to have gone home sooner ○ Stay was the right length ○ Prefer to have stayed longer
